# On the correlation between gratitude and resilience in medical students

**DOI:** 10.3205/zma001663

**Published:** 2024-02-15

**Authors:** Nicolai Hahn, Patrick Brzoska, Claudia Kiessling

**Affiliations:** 1 Witten/Herdecke University, Faculty of Health, Education of Personal and Interpersonal Competencies in Health Care, Witten, Germany; 2 Witten/Herdecke University, Faculty of Health, Health Services Research, Witten, Germany

**Keywords:** gratitude, resilience, optimism, resilience factors, medical studies, mental health

## Abstract

**Objective:**

Medical students’ health and resilience have increasingly been the subject of current research in recent years. A variety of interventions are recommended to strengthen resilience or its known or suspected influencing factors, although the literature shows that the evidence on the effectiveness of the interventions is inconsistent. The present study investigated whether gratitude is a direct protective factor for resilience in medical students or whether resilience factors (optimism, self-efficacy, social support) and stress mediate the effects of gratitude on resilience.

**Methods:**

90 medical students at Witten/Herdecke University took part in the study that determined their gratitude, resilience, optimism, self-efficacy, social support and stress levels using validated questionnaires (GQ-6, RS-25, LOT-R, SWE, F-SozU, PSS). Correlations were analyzed using Pearson correlation coefficients. In addition, a multivariate regression analysis and a path analysis were calculated to determine the direct and indirect effects of gratitude on resilience.

**Results:**

Multivariate regression analysis showed that only optimism, social support and stress were significantly associated with resilience (B=0.48, 95% CI: 0.31, 0.66; B=0.23, 95% CI: 0.01, 0.44 and B=-0.02, 95% CI: -0.03, -0.001, respectively). The direct effect of gratitude on resilience was minimal and not significant in the path analysis. However, there was an indirect effect of gratitude on resilience (B=0.321; p<0.05). Mediation via the optimism variable was mainly responsible for this effect (indirect effect B=0.197; p<0.05).

**Conclusion:**

This study shows that gratitude has only a minimal direct influence on resilience. However, results indicate that optimism as a mediating factor strengthens the resilience of medical students. Against this background, it may be useful to integrate interventions that promote an optimistic attitude into medical studies in order to strengthen the mental health of future doctors in the long term.

## Zusammenfassung

**Zielsetzung:**

Die Gesundheit und Resilienz von Medizinstudierenden sind seit einigen Jahren zunehmend Gegenstand der aktuellen Forschung. Zur Stärkung der Resilienz oder ihrer bekannten oder vermuteten Einflussfaktoren werden eine Vielzahl von Interventionen empfohlen, wobei die Literatur zeigt, dass die Evidenzlage zur Wirksamkeit der Interventionen uneindeutig ist. Die vorliegende Studie untersuchte, ob Dankbarkeit ein direkter Schutzfaktor für Resilienz bei Medizinstudierenden ist oder ob Resilienzfaktoren (Optimismus, Selbstwirksamkeit, soziale Unterstützung) sowie Stress Effekte von Dankbarkeit auf Resilienz vermitteln.

**Methodik:**

An der Studie nahmen 90 Medizinstudierende der Universität Witten/Herdecke teil, deren Dankbarkeit, Resilienz, Optimismus, Selbstwirksamkeit, soziale Unterstützung und Stresslevel mit Hilfe von validierten Fragebögen (GQ-6, RS-25, LOT-R, SWE, F-SozU, PSS) ermittelt wurden. Korrelationen wurden mittels Pearson-Korrelationskoeffizienten analysiert. Zusätzlich wurden eine multivariate Regressionsanalyse und eine Pfadanalyse berechnet, um direkte und indirekte Auswirkungen von Dankbarkeit auf Resilienz zu ermitteln.

**Ergebnisse:**

Die multivariate Regressionsanalyse zeigte, dass lediglich Optimismus, soziale Unterstützung und Stress signifikant mit Resilienz assoziiert sind (B=0,48, 95%-KI: 0,31-0,66; B=0,23, 95%-KI: 0,01; 0,44 bzw. B=-0,02, 95%-KI: -0,03; -0,001). Der direkte Effekt von Dankbarkeit auf Resilienz war in der Pfadanalyse minimal und nicht signifikant. Es zeigte sich allerdings ein indirekter Effekt von Dankbarkeit auf Resilienz (B=0,321; p<0,05). Für diesen Effekt war hauptsächlich die Mediation über die Variable Optimismus verantwortlich (indirekter Effekt B=0,197; p<0,05).

**Schlussfolgerung:**

Die vorliegende Studie zeigt, dass Dankbarkeit nur einen minimalen direkten Einfluss auf Resilienz hat. Die Ergebnisse weisen allerdings darauf hin, dass Optimismus als vermittelnder Faktor die Resilienz von Medizinstudierenden stärkt. Vor diesem Hintergrund könnte es sinnvoll sein, Interventionen, die eine optimistische Grundhaltung fördern, ins Medizinstudium zu integrieren, um damit die mentale Gesundheit zukünftiger Ärzt*innen langfristig zu stärken.

## 1. Introduction and objectives

It has been known for decades that stress and strain during medical studies can lead to states of exhaustion and serious illnesses [1], [2], [3]. Medical students worldwide are, for example, more than twice as likely to suffer from depression or depressive symptoms compared to the general population [4], [5]. This also applies to medical students in Germany [6], [7]. Research findings also indicate that medical students are exposed to higher levels of psychological stress than students of other disciplines [8], [9], [10]. Further data shows that mental health deteriorates during medical studies and continues to decline on average when students enter professional life [11], [12], [13]. Physicians’ mental health usually does not stabilize with increasing professional experience, so that the prevalence of depression, stress and suicide is also higher in this group than in the general population [14], [15], [16]. It is also known that medical doctors’ mental health has a direct impact on patients’ health [17]. Therefore, it seems sensible to find ways to positively influence students’ mental health as early as during medical school. The concept of resilience could play a key role here. Resilience refers to the ability to successfully adapt to acute stress, trauma or chronic stress and to remain healthy despite negative stressors, such as stressful events [18], [19], [20]. Studies have shown that people with a high level of resilience are less depressed, fall ill less often and also recover more quickly after illness than people with a low level of resilience [18], [19], [21], [22], [23]. Resilience is considered to be something that can be learned, changed and trained throughout life [24], [25], based on the neuroplasticity of the human brain [26]. Against this background, interventions that can strengthen resilience are of particular interest, as they can make a lasting contribution to maintaining health. However, according to a Cochrane Review, interventions that aim to strengthen resilience show inconsistent evidence regarding their effectiveness [27]. On the other hand, promoting so-called resilience factors can have an effective influence on resilience [21], [24], [27]. These are psychological and social resources that have a positive effect on resilience [28]. They include, for example, the experience of positive emotions [29], optimism [24], hope [30], a sense of coherence [31], [32], self-efficacy [33], and social support [28]. Resilience factors in medical students have also been investigated in a number of studies [34], [35], [36]. These resilience factors are closely related to the concept of gratitude, as gratitude can have a positive influence on resilience factors [37], [38], [39], [40], [41]. Gratitude has only been systematically investigated in medical-psychological research for around 20 years, and there are various definitions of gratitude [42]. For example, gratitude is conceptualized as an emotion, attitude, virtue or character trait [43]. One of the most widely used definitions and conceptualizations of gratitude comes from the American psychologists Michael McCullough and Robert A. Emmons. According to them, gratitude means perceiving the benevolence of another person or the environment and reacting to the resulting experience with positive emotions [37]. 

Assuming that resilience factors have a protective effect on resilience and that gratitude in turn has positive effects on these resilience factors, the present study aims to test the hypothesis that gratitude acts as a predictor of resilience in medical students and that the resilience factors optimism, self-efficacy expectation and social support mediate indirect effects of gratitude on resilience. These resilience factors were selected because they are considered to be very well researched and also cover the spectrum of personal and interpersonal protective factors [24]. Although individual studies show that gratitude is a predictor of resilience [44], [45], it remains unclear whether the results are also transferable to other populations or whether the effect is mediated by other variables.

## 2. Methodology

### 2.1. Setting 

We conducted a cross-sectional survey with medical students at Witten/Herdecke University (UW/H) in the preclinical phase of their studies (semesters 2-4). They were approached personally in seminars and asked to participate voluntarily via the internal e-mail distribution list. Of 176 possible participants, 94 took part in the study (response rate=53.4%). The survey took place in December 2019 and June 2020. The study was reviewed and approved by the ethics committee of Witten/Herdecke University (application number: 187/2019). 

### 2.2. Instruments 

Gratitude was measured using the German version of the Gratitude Questionnaire-6 (GQ-6) [37], [46], one of the most frequently used questionnaires to survey this construct [47]. It consists of six items, each with a seven-point response format ranging from 1 (strongly disagree) to 7 (strongly agree). Mean values were calculated (values between 1 and 7). The original scale has a high internal consistency, with a Cronbach’s alpha of 0.82 [37]. 

The German translation of the RS-25 resilience scale by Wagnild & Young [48] was used to measure resilience [49]. The scale has shown to be a valid instrument for measuring resilience [50] and is the most widely used scale to assess resilience [21]. The instrument comprises 25 items, which are rated from 1 (No, I disagree) to 7 (Yes, I completely agree). The resilience scale is divided into two subscales: “personal competence” (17 items) and “acceptance of self and life” (8 items) [51]. The total scale was used in this study (mean values between 1 and 7). The internal consistency of the German Resilience Scale (RS-25) is high, with a Cronbach’s α=0.94 [49]. 

The German revised version of the Life Orientation Test (LOT-R) was used to measure the tendency towards dispositional optimism [52]. The Life Orientation Test contains a total of ten items: three positively formulated items in terms of optimism; three negatively formulated items in terms of pessimism and four filler items; all items have a seven-point response format ranging from “strongly disagree” (1) to “strongly agree” (7). In the present study, mean values were calculated for the assessment of optimism (values between 1 and 7). The internal consistency of the German version of the Life Orientation Test is α=0.59 for the overall scale [52].

Self-efficacy expectations were measured using the General Self-Efficacy Scale (SES) [53]. The questionnaire comprises ten items, which are rated from 1 (strongly disagree) to 4 (strongly agree). Mean values were calculated (values between 1 and 4). The scale for measuring SES shows an acceptable internal consistency of Cronbach’s α=0.78 [53].

The questionnaire on social support (F-SozU; [54]) was used to determine the level of social support. The short form of the questionnaire was used (K14; [55]), which consist of 14 items with a five-point response format from 1 (Does not apply) to 5 (Applies exactly) (mean values between 1 and 5). The short form (K14) of the questionnaire on social support shows very good internal consistency with a Cronbach’s alpha of 0.94 [55]. 

Stress was measured using the German translation of the Perceived Stress Scale (PSS) [56], [57]. The questionnaire comprises ten questions, which are assessed by means of a five-point response format from 1 (never) to 5 (very often). The stress scale is divided into two subscales, the “helplessness” scale on the one hand and the “self-efficacy” scale on the other. In the present study, total values were calculated (values between 10 and 50). The internal consistency of the stress scale is high and lies between α=0.79 and 0.89 [57].

### 2.3. Data analysis

Individual questionnaires were first analyzed descriptively at the level of the overall scales. In addition, the internal consistency (Cronbach’s alpha) for the study population was determined for each scale (see table 1 (Tab. 1)). To answer the research questions, a bivariate correlation (Pearson’s correlation coefficients) and a multivariate regression were calculated [58]. A path analysis was carried out to examine the indirect effects of gratitude on resilience [59], [60]. The significance level was set at α=0.05 for all analyses. Data analysis was performed using the IBM Statistics SPSS program version 26 and R/lavaan 0.6-3 [61]. Four questionnaires were incomplete or incorrectly filled in. These values were not included in the data analysis. 

## 3. Results

### 3.1. Sample and descriptive statistics

The study included 90 medical students, of these 30 were male (33.3%) and 60 were female (66.7%). Descriptive statistics of the individual scales are shown in table 1 (Tab. 1). Internal consistencies (Cronbach’s α) are high in each case and lie between 0.78 and 0.90. Only the “optimism” scale has an internal consistency of α=0.62, which can be considered comparatively low.

### 3.2. Correlation analysis

Bivariate Pearson correlation coefficients of the scales are shown in table 2 (Tab. 2). Both gratitude and resilience correlated weakly to moderately with all other scales included in the study. 

### 3.3. Multivariate regression analysis 

A multivariate regression was performed to examine how strongly the variables gratitude, optimism, self-efficacy expectation, social support, and stress predict resilience in the study population. The model statistically significantly predicted resilience (F(5.84)=22.44, p<0.001, R^2^=0.572). The variables optimism, social support, and stress each contributed statistically significantly to the prediction (p<0.05). Gratitude showed only a minimal and non-significant effect on resilience. Optimism showed the strongest significant correlation with resilience (see table 3 (Tab. 3)). The inclusion of gender as a confounder showed no significant effect on resilience and had no effect on the correlation of the other variables.

### 3.4. Path analysis

**Figure 1:**
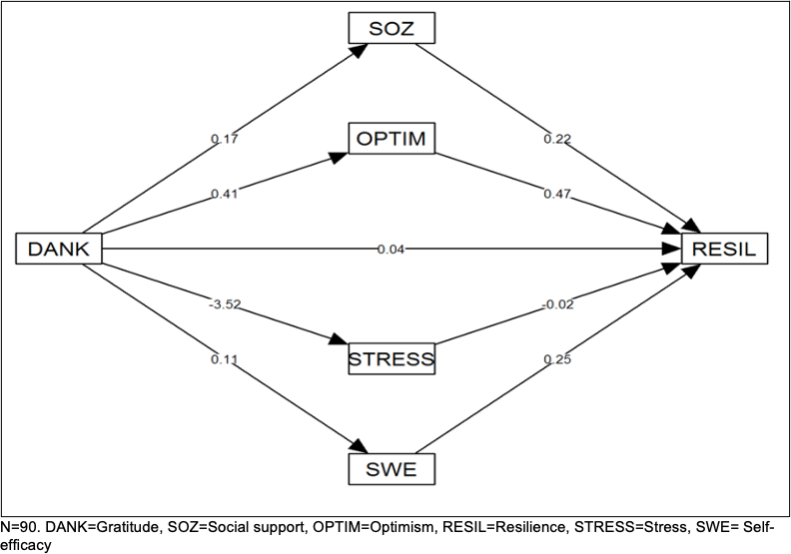
Path model analysis to test the hypothesis that there is an indirect effect of gratitude on resilience. Cross-sectional survey of medical students

A path analysis was also carried out to examine whether the effect of gratitude on resilience is overlaid by other variables and whether gratitude has an indirect effect on resilience via resilience factors or (reduced) stress. In this analysis, gratitude was selected as the independent variable, resilience as the dependent variable and optimism, self-efficacy expectation, social support and stress as mediators. The results showed that the indirect effect of gratitude on resilience – mediated by social support, optimism, stress and self-efficacy – (total indirect effect B=0.315) is statistically significant (p<0.05). It was found that the variable optimism was mainly responsible for this effect (indirect effect B=0.192, p<0.05). All other variables were not statistically significant. This result suggests an indirect effect of gratitude on resilience, mainly mediated by the variable optimism. Adjusting for gender had little overall impact on the effects (see figure 1 (Fig. 1)).

## 4. Discussion

### 4.1. Summary of the most important results 

The aim of the study was to investigate whether gratitude has a direct effect on the resilience of medical students in the sense of an independent resilience factor or whether it has an indirect effect on resilience via other resilience factors such as optimism, self-efficacy expectations, social support, or stress. Previous studies have not sufficiently clarified this relationship, as the relationship between gratitude and resilience has been investigated and confirmed, but other resilience or confounding factors were not included in the respective studies [44], [45]. The present study showed a positive correlation between gratitude and resilience. The subsequent regression analysis showed that 57% of the total variance in resilience can be explained by the variables gratitude and the resilience factors optimism, self-efficacy expectation, and social support as well as stress. Of these, optimism had the only significant and strongest direct influence on resilience. The direct effect of gratitude on resilience is negligible, which means that gratitude cannot be regarded as a direct predictor of resilience among the medical students in this study. Possible explanations for this result are that the few studies showing that gratitude is a predictor of resilience used a different resilience scale or did not take competing variables (resilience factors) into account [44], [45]. However, the path analysis showed that there is an indirect effect of gratitude on resilience and that the relevant parameter is again optimism. Optimism therefore has a direct effect on resilience on the one hand and an indirect effect on the other hand by mediating the effects of gratitude on resilience. 

The results of the present study are consistent with findings from research into the relationship between optimism and resilience. An optimistic attitude has long been associated with other positive factors, such as physical and psychological well-being, lower levels of depression, and greater life satisfaction [62], [63], [64]. Both gratitude and resilience are closely related to optimism [65], [66]. In addition, optimism also appears to be a predictor of resilience [67], [68]. The present study now shows that this correlation can also be assumed for medical students. 

The mean values of medical students for gratitude in this study are high. Studies in which students were also surveyed, albeit of other disciplines, show similarly high or only slightly lower values [28], [69], [70]. The mean values for resilience of the medical students in this study are lower than the values of students of other disciplines [71] and the German population in the age group from 14 to 30 years [51]. There are numerous possible reasons why the resilience scores of medical students are lower than those of other students or other study populations. Surveys show, for example, that medical students are exposed to high levels of stress [2], [72], that they more often suffer from sleep disorders [73], and from depression [4], [5]. The type of medical program (standard or reform program) also appears to have an influence on students’ stress levels [74]. Last but not least, the COVID-19 pandemic has also led to stress and uncertainty among medical students [75], [76], [77]. All these factors can have a negative impact on medical students’ resilience. The mean values for optimism in the present study are slightly higher than in comparable studies that also examined medical students [34], [78], [79]. One reason for this may be that only medical students at the beginning of their studies were surveyed here, whereas the comparable studies surveyed medical students of all year groups [78], [79] or only at an advanced stage [34]. It seems plausible that medical students’ optimism decreases over the course of their studies, as their general mental health also deteriorates [11], [12], [13].

In summary, it can therefore be stated that gratitude has no significant direct effect on resilience in medical students. The initial hypothesis that gratitude is a predictor of resilience must therefore be rejected. However, the second hypothesis that there are indirect effects of gratitude on resilience that are mediated by resilience factors was confirmed. This shows that optimism in particular strengthens medical students’ resilience.

### 4.2. Possible implications for medical education

Knowledge of the positive effects of an optimistic attitude on medical students’ resilience can support the design and implementation of interventions aimed at positively influencing medical students’ resilience. Approaches such as the “Best Possible Self” (BPS) writing intervention developed by Laura King are interesting in this context. In this exercise, participants write about themselves in the future and imagine that their future life will turn out in the best possible way [80]. The BPS was examined with regard to its effect on a wide range of factors [81]. It was demonstrated that the BPS can also be used to promote an optimistic attitude [82], [83], [84]. Particularly in view of the limited evidence for resilience interventions that directly target resilience [27], the possible effect of an optimistic attitude on medical students’ resilience is becoming increasingly important. It may therefore be worthwhile to develop interventions that aim to strengthen students’ optimism in order to achieve a positive effect on their resilience and thus promote their mental health. In addition to interventions aimed at strengthening optimism, there is also a large number of gratitude exercises with effects on various outcome variables [85], [86], [87]. However, the evidence base for gratitude interventions is rather weak [88]. This could be explained by the overall low direct effects of gratitude on resilience, which the present study also points out. The study shows that it may be useful in future research on gratitude exercises to include optimism as a dependent outcome variable, as it is possible that gratitude training strengthens optimism, which in turn has a positive influence on resilience. 

### 4.3. Limitations of the study

The present study has some limitations. Firstly, the sample size was relatively small. In addition, only students at one university were recruited, which is why it would be interesting to repeat the study at other locations. Furthermore, only pre-clinical students were examined, so the results can only be applied to a certain proportion of medical students. Possible changes during the course of studies can therefore not be mapped. The fact that the age of the participants was not recorded also leaves open to which extent age influenced the results. Moreover, all values collected are based on self-assessments and are therefore subjective. In order to make resilience research more objective, some researchers extend the self-assessment via questionnaires by also measuring the cortisol levels of test subjects [89]. It will certainly remain a challenge and the subject of future research to approach constructs such as gratitude and resilience appropriately in terms of objectification and operationalization. While the scales for measuring resilience, stress, self-efficacy expectation, social support and gratitude showed high internal consistencies comparable to other studies [37], [49], [53], [55], [57], the internal consistency of the scale for measuring optimism (Life Orientation Test) was comparatively low. This is not surprising given that a similarly low internal consistency (α=0.59) was observed in the validation study of the Life Orientation Test. This may indicate general methodological problems with the scale and shows the need for further methodological development in this area. The extent to which the limited internal consistency of the scale influenced the results of the present analysis remains unclear. 

More women than men took part in the study. One explanation for this may be that significantly more women than men study medicine in Germany. According to the Federal Statistical Office, 37,036 of 98,736 medical students in 2019 were male (≈38%) and 61,700 were female (≈62%) [https://www.destatis.de/EN/Themes/Society-Environment/Education-Research-Culture/Institutions-Higher-Education/_node.html]. These figures are similar to the gender ratio at Witten/Herdecke University (male: 41% vs. female: 59%) [https://ranking.zeit.de/che/de/studiengang/6115] and also to the gender ratio in the present study. In addition, studies show that women generally appear to be more likely to participate in health surveys than men [90], [91]. It is also conceivable that women feel more attracted to a study on gratitude and resilience than men; however, this remains an assumption, as there is insufficient research on this topic. However, other studies on gratitude or resilience also show a similar gender ratio [27], [28]. The influence of other variables such as native language, nationality and religious affiliation was not investigated and should be the subject of future research. In addition, results may have been distorted as the survey was designated as a study on resilience and gratitude. This could, in the sense of a selection bias, have appealed to students who had already dealt with the respective concepts. 

## 5. Conclusions

This study shows that gratitude has no significant direct effect on resilience. However, it was shown that optimism can strengthen the resilience of medical students and that gratitude has a mediated effect on resilience via optimism. The relationship between resilience and resilience factors therefore appears to be complex, meaning that a number of influencing factors and their interactions must be taken into account when providing trainings to strengthen resilience. The general conditions of studying and working must also be taken into account. Resilience helps people to stay healthy or to recover more quickly after challenges. Against this backdrop, it could make sense to integrate interventions that promote an optimistic attitude into medical studies and thus strengthen the mental health of future doctors in a preventive and long-term manner. Which interventions are particularly suitable for medical students must be further investigated in future controlled and prospective studies. The results of this study can be used to better understand the underlying mechanisms that contribute to medical students’ resilience and the role that optimism plays in this.

## Authors’ ORCIDs

- Nicolai Hahn: 0009-0003-5363-0516
- Patrick Brzoska: 0000-0001-6489-5198
- Claudia Kiessling: 0000-0003-4104-4854

## Acknowledgements

The authors would like to thank all students of the Faculty of Health at the UW/H who participated in this study and Christina Wagner for her support in translating the English version of the article.

## Competing interests

The authors declare that they have no competing interests. 

## Figures and Tables

**Table 1:**
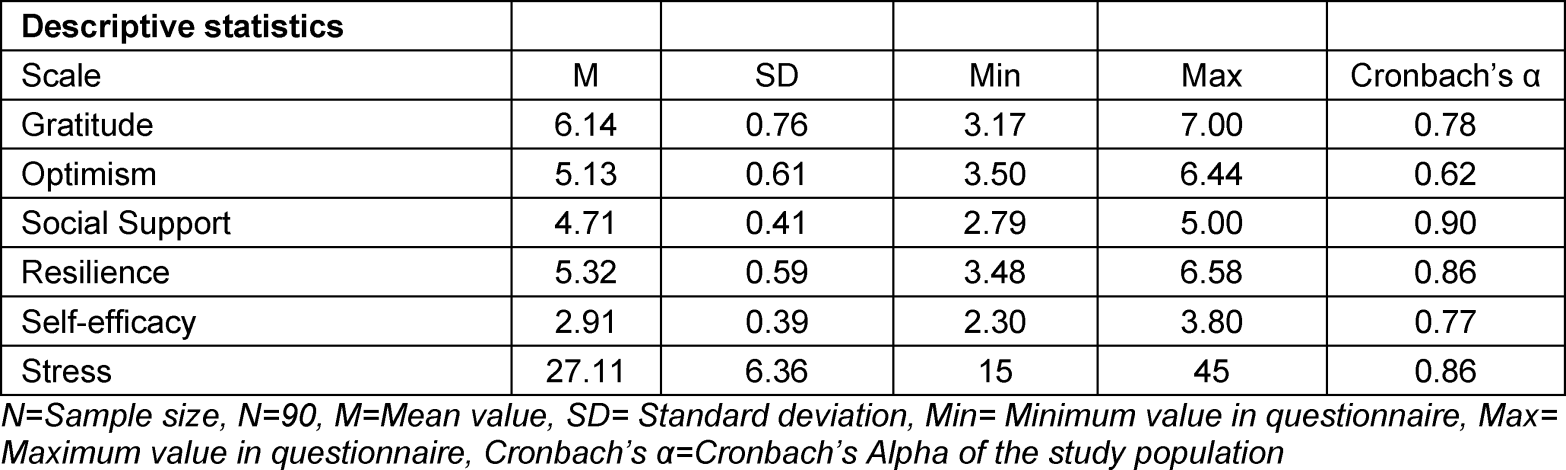
Descriptive statistics of the constructs considered in the study

**Table 2:**
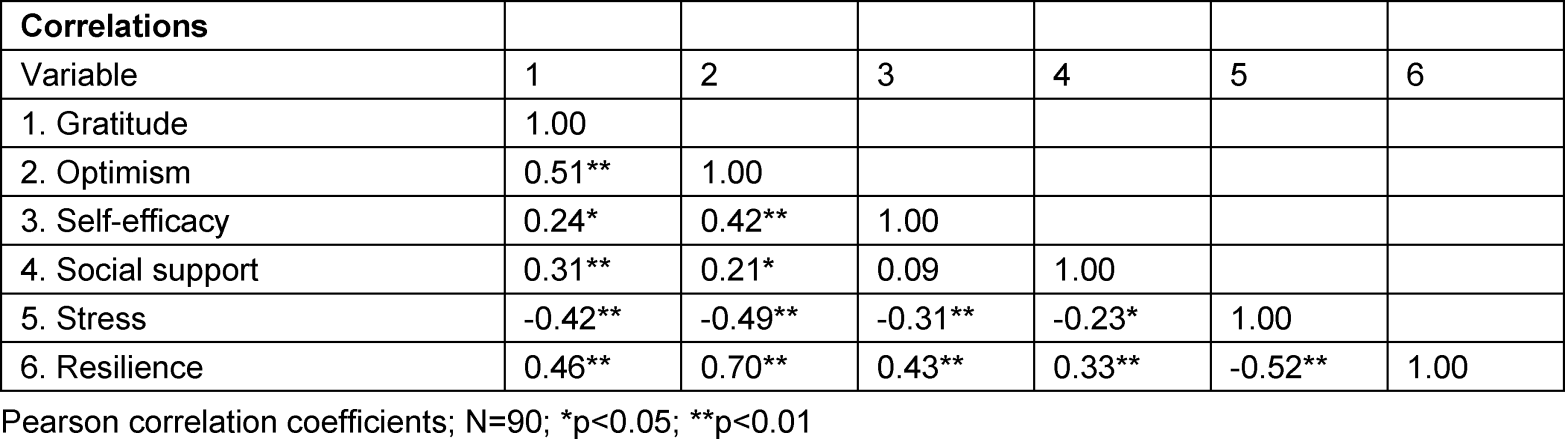
Correlation analysis of the constructs considered in the study

**Table 3:**
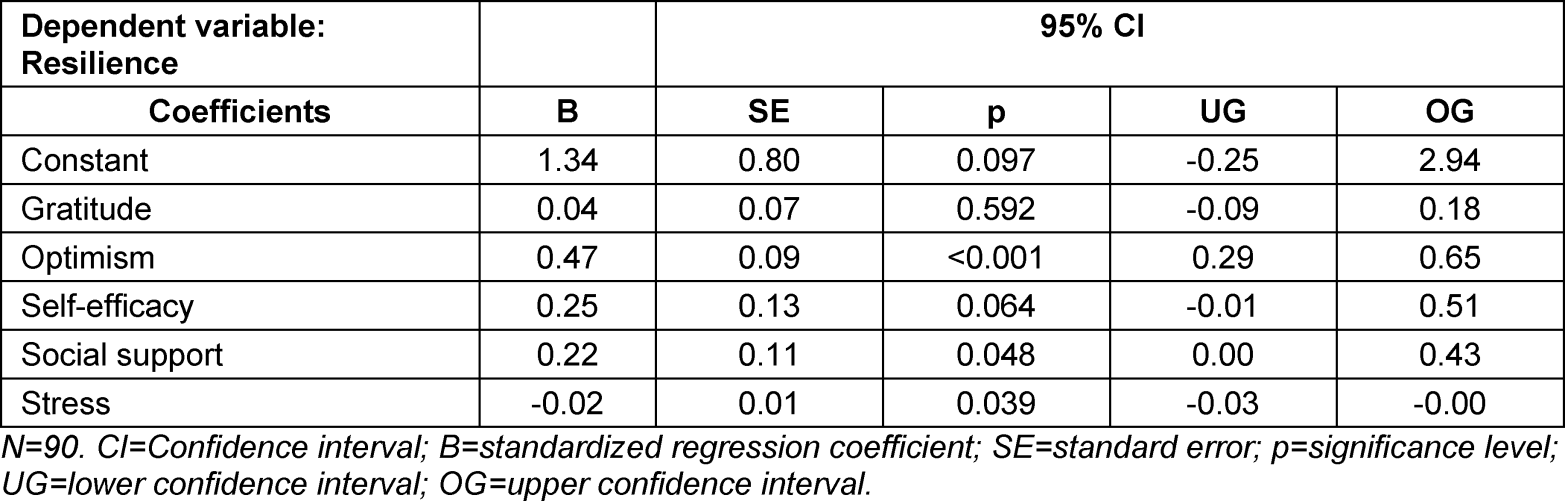
Multivariate regression analysis to test the hypothesis of whether gratitude is a predictor of resilience in medical students
